# New Products

**DOI:** 10.1155/S1463924684000444

**Published:** 1984


Journal of Automatic Chemistry, Volume 6, Number 4 (October–December 1984), pages 216–220

### New Products

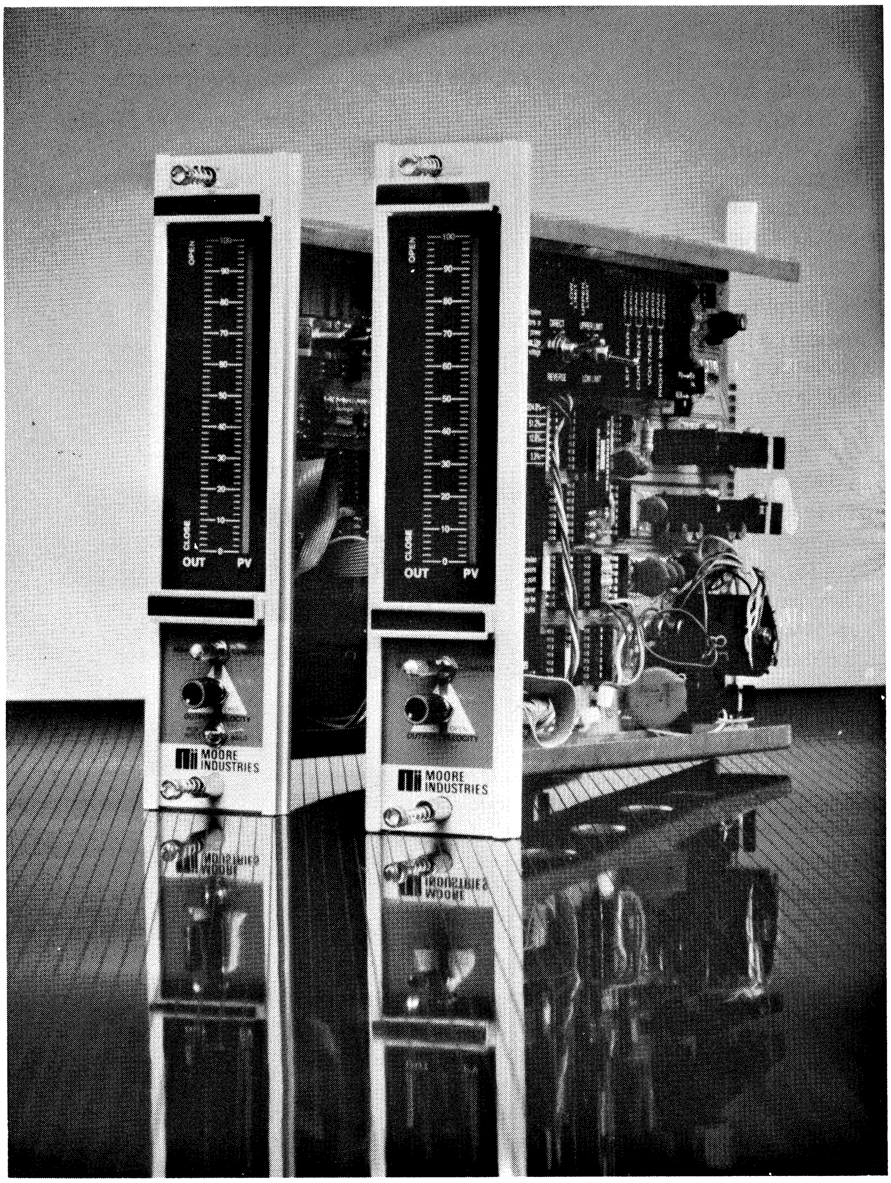

*Moore Industries' DAI (direct analogue interface). The DAI's design ensures that accidental adjustments do not occur and even if it is removed or subjected to power-failure when in the computer-control mode, the process is not interrupted.*

### Computer interface for safeguarding processes

Moore Industries Europe have introduced an interface which safeguards the correct functioning of a final control element in the event of failure of the PC or computer. It also ensures bumpless transfer to manual or computer mode.

Called the DAI (direct analogue interface) the new unit is available either as a single loop fail-safe back-up or as a single-channel demultiplexer.

As a back-up unit, the DAI interfaces the PC or computer with the final control element. In the event of PC or computer failure the DAI holds the last level of control signal to the control element and LEDs on the panel indicate that the unit has assumed manual control. The operator can then manually adjust the control signal until the fault is rectified and subsequently transfer bumplessly to computer mode.

The unit may be programmed to give any output required when first powered-up and this facility is particularly useful in speeding up plant 'start-up'.

As a demultiplexer, a number of DAIs can be interfaced with a single analogue output of a computer or PC. Each DAI is updated by the computer to manipulate several final control elements.

Two bar-graph displays on the front of the unit show the output from the DAI and the process variable under control. In manual mode, these displays are used to monitor the process signal allowing adjustments of the DAI output to be made in accordance with the requirements of the process. Feedback to the computer enables its output to move to match the output from the DAI when transferring back to computer mode.

In addition, one of the bar graphs will display an auxiliary signal on a temporary basis by means of a 'hold on' switch: this auxiliary input is commonly used to display the computer output signal.

Both displays double as alarms, flashing at a fixed frequency of 2 Hz for overrange indication and double that frequency for a true alarm condition. Alarms are initiated by external contact closures.

*Full specification from Moore Industries Europe, Baird Close, Maxwell Way, Crawley, West Sussex RH10 2SY, UK. Tel.: 0293 514488.*

**Circle No. 153 on Reader Enquiry Card**

### NTIS data-base

Three National Technical Information Service (US Department of Commerce) Published Searches are now available in the UK from Microinfo. They are:

*Toxicity of Manganese (PB84-869510)*: an updated bibliography containing 181 citations taken from between 1970 and June 1984. Topics covered concern the toxicity, carcinogenicity, environmental pollution and other hazards and adverse effects of manganese. The detection, characterization, analytical methods, standards and removal from the environment are considered. These aspects of manganese are dealt with in relation to aquatic and terrestrial flora and fauna, including man. Manganese pollution from mining operations is also discussed.

*Toxicity of Zinc* (*PB84-869593*) contains 329 citations concerning health effects related to the uptake and toxicity of zinc. The emissions of trace elements of zinc in the atmosphere, the sources of these emissions, monitoring techniques and accumulation in the environment are topics which are considered in the bibliography. Citations cover the period from 1970 to June 1984.

*Toxicity of Nickel* (*PB84-868991*) discusses the toxicity, carcinogenicity, environmental pollution and other effects of nickel, including the detection, characterization, analytical methods, standards and removal of this metal from the environment. This bibliography contains 184 citations and again presents material published between 1970 and June 1984.

*Details from Microinfo Ltd, PO Box 3, Newman Lane, Alton, Hampshire GU34 2PE, UK. Tel.: 0420 86848.*

**Circle No. 154 on Reader Enquiry Card**

### Application on cassette

The application of an advanced distillation-control package is explained on an audio-cassette tape. The 28 min recording was prepared by Houston-based Setpoint's separation control staff.

Beginning with an overview of distillation control, the tape identifies the various ways advanced control can be applied to single and multiple columns, and how optimization is used to increase profits. The tape then gives details of these strategies, describes the common operating problems encountered, and shows how Setpoint's approach can resolve them. Most of the recording is devoted to a discussion of the technical aspects of the package, with emphasis on how it uses key product values to achieve optimal benefits from a distillation train.

*For a free copy, write to Setpoint, Inc., 950 Threadneedle, Houston, Texas 77079, USA or phone 713 496 3220.*

**Circle No. 155 on Reader Enquiry Card**

### East Anglia Chemicals

Sanderson Holdings has bought the goodwill, assets and stock of East Anglia Chemicals—Sanderson intend to expand EAC's business as a supplier of laboratory, electronic and fine chemicals. The production plant of stainless-steel, glass and glass-lined equipment covers reaction, blending, vacuum filtering, centrifuging, fluid bed and chamber drying, together with glass vacuum distillation units for high-purity pharmaceutical products. Commodity supplies are available from normal laboratory sizes to bulk lots in the order of 1000 kilo-litres. These are production and technical facilities available to suit customers specifications or custom-processing requirements.

*Information from EAC at Hadleigh, Ipswich IP7 6BQ, UK. Tel.: 0473 823083.*

**Circle No. 156 on Reader Enquiry Card**

### ACA V (Du Pont)

Du Pont has announced a new clinical instrument, the ACA V discrete clinical analyser. The ACA V provides faster test results and improved data for clinical laboratories, whilst retaining the key features of the ACA system concept: ease of operation, random access, 24 h availability, use of pack chemistries and an expandable test menu.

Highlighted features of the ACA V include providing first test results in 5·5 min (1 min faster than previous models) and subsequent results every 37 s, as well as incorporation of an ion-selective electrode (ISE) which increases throughput and requires smaller samples. ISE tests are used for procedures such as sodium (Na) and potassium (K) assays and do not require test packs.

It has a two-piece sample system, 'sufficient sample' detection (eliminating erroneous results from too small samples) and a larger display screen for results. The ACA also provides a wider report slip and has easy-access clue cards, a drop-down shelf (for easier handling of diluent containers), and extra alarms.

Future improvements include additional electrodes and memory, and data-management software. It is expected that by mid 1985 electrodes for Cl and $TCO_2$ will be added permitting a higher throughput and cutting test costs. Introduction of data-management software, planned for late 1985, will be optional. Its proposed applications will include: auto-calibration, quality and inventory control, report formatting, result and work-load reporting, and preventive maintenance records.

The range of 59 fully-automated tests now available covers general and special chemistries, enzymes, endocrine functions, toxicology, electrolytes, immunology, coagulation tests, and therapeutic drug monitoring including the anti-arrythmic digoxin. The ACA V can run as many as 180 tests/h. There are at present more than 4000 Du Pont analysers in use world-wide.

*For further information contact: R. S. Iredale, Du Pont de Nemours International S.A., PO Box, 1211 Geneva 24, Switzerland. Tel.: 022/37 87 03.*

**Circle No. 157 on Reader Enquiry Card**

### Evaluation of microbiological analyser

The Bactec (Becton Dickinson Laboratory Systems) 460 Automated Microbiological Analyser has had a favourable evaluation report by the DHSS's Scientific and Technical Branch. The evaluators (J. Farrar and A. Paull, University Hospital Wales, and J. Boyce and R. Powell, East Glamorgan Hospital) used separate Bactecs at each location for a study involving over 3000 patient samples. They investigated all the operational and financial aspects of running the instrument including storage, handling and disposal of vials, ease of operation, reliability, reproducibility of results, cross-contamination levels and the amount of technician time required to operate the instrument. These were compared to the conventional blood culture protocols formerly used at each laboratory. In addition to these exhaustive tests they also carried out a series of sensitivity checks using standard dilutions of known organisms. Also included is a report on tests carried out to measure any environmental contamination arising from use of the Bactec, undertaken by CMSRL at Porten Down. All three reports are thorough and their conclusions very favourable; East Glamorgan stating that they would have 'no hesitation in recommending the instrument for use in diagnostic laboratory work'.

*This report is now available from the Department of Health and Social Security, 14 Russell Square, London WC1B 5EP (Project officer: A. Horn). Further details of the Bactec 460 system from Laboratory Impex Ltd, Lion Road, Twickenham, Middlesex, UK. Tel.: 01 891 4881.*

**Circle No. 158 on Reader Enquiry Card**

### AA data-management system

A new software package allows the Varian DS-15 computer to be linked to the AA-1275 or AA-1475 spectrophotometer for complete atomic absorption data management. The DS-15 is a desk-top computer with 12 in CRT, 128 K bytes RAM and two disk drives providing 720 K bytes storage. The single-beam AA-1275 and double-beam AA-1475 atomic absorption spectrophotometers have a good reputation for performance, reliability and ease of use. The new Varian software brings these together in a package specifically tailored to meet the needs of the busy analytical laboratory. No programming experience or typing ability is required. The operator is guided through a series of simple selections using menus and soft keys. Results are collected automatically when flame or furnace autosamplers are used; samples may also be presented manually.

Flexible report generation allows reports to be tailored to individual needs. The operator may choose the amount of data to be printed and the format of the report. Data from flame, furnace and hydride analyses may be combined in a single report. Analytical data can be permanently stored in a disk-based archive file. Data editing allows any spurious reading to be removed: this is particularly useful with graphite furnace work.

The graphics program (see photograph) displays background and corrected signal, providing an invaluable aid for furnace method development.

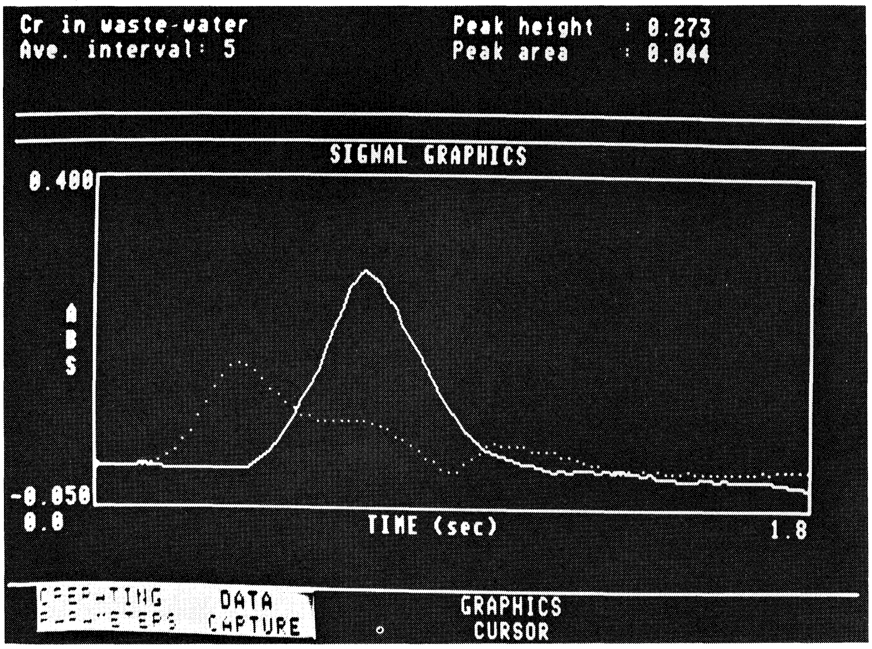

*Generated by Varian's new software package. A combination of a DS-15 computer and Varian's AA spectrophotometers, the system is described as improving productivity by freeing laboratory staff for tasks other than data processing.*

*More information from Varian Associates Ltd, 24–28 Manor Road, Walton-on-Thames, Surrey, UK or Varian AG, Steinhauserstrasse, CH 6300, Zug, Switzerland.*

**Circle No. 159 on Reader Enquiry Card**

### Sample processor

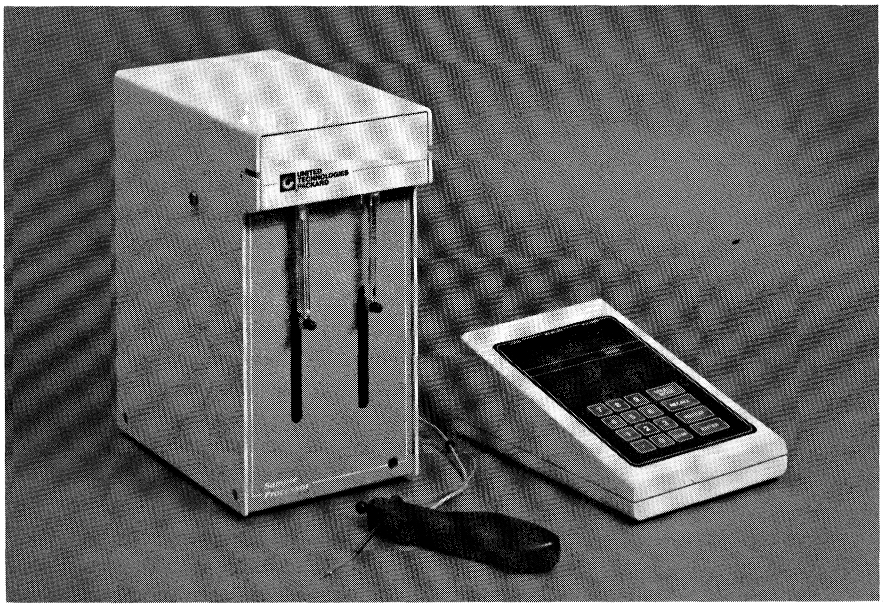

*Model 1700 sample processor. The hand probe provides control of the initiation of each cycle. (Packard Instrument Ltd, UK.)*

The Packard Model 1700 Sample Processor is a programmable, digital diluter. There are two modules, the diluter and the keyboard, which perform such functions as pipetting, diluting, dispensing and multiple dispensing, sample-only transferring, sample aliquoting, serial diluting and titrating.

The diluter is driven by a Z-80 microprocessor and can communicate with other instruments via an RS232 port.

The keyboard is a 12-button keypad with four command keys; a touch-sensitive membrane overlay protects the keyboard from chemical spills. An eight-character alphanumeric vacuum fluorescent display shows program information, memory location, and error codes. Below it, a blackout display shows operating modes and phases. The movable keyboard module is attached to the sample processor by a flexible cord and can be positioned to maximize operator convenience.

The instrument will accomodate syringes of various sizes: the aspirate syringe pipettes sample or reagent, and the reagent syringe draws in the reagent that is to be dispensed. At the end of a test, a 'reagent-save' feature dispenses reagent left in the reagent syringe back into the reagent vessel.

The diluter includes a removable syringe-cassette, which houses all the components that come in contact with the reagents, including valves, tubes and syringes. This feature allows a change from one procedure to another in seconds and the use of radioactive or other toxic liquids without risk of cross-contamination.

*Further information from Packard Instrument Ltd, 13–17 Church Road, Caversham, Reading, Berkshire RG4 7AA, UK. Tel.: 0734 478234.*

**Circle No. 160 on Reader Enquiry Card**

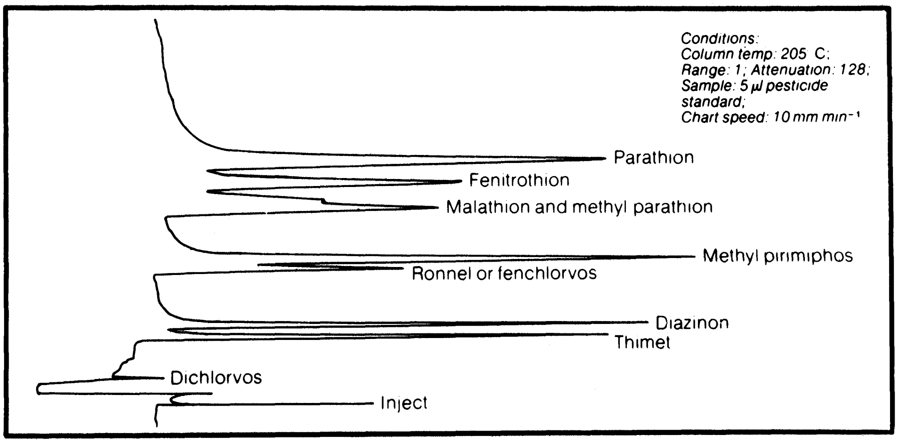

*Investigations by Pye Unicam's applications chemists have proved that the nitrogen detectors used in the company's gas chromatograph systems will respond favourably to organo-phosphorus compounds. The design of the nitrogen detector—which is based on alkali flame ionization principles—and the nature of the salt tip used (RbCl), were originally optimized to give a maximum response towards organo-nitrogen compounds. The suggestion that the detector, without modification, would also respond to organo-phosphorus compounds, came from customers. The chromatogram of a mixture of pesticides, illustrated here, shows how promising the results were. Following this encouraging start, further investigation showed that a detectability of $2 \times 10^{-15}$ gP/s could be obtained by a simple optimization procedure, using the standard rubidium chloride tips. (Pye Unicam Ltd, York Street, Cambridge CB1 2PX, UK; tel.: 0223 358866.)*

**Circle No. 161 on Reader Enquiry Card**

### OEM economy thermometer

A dial thermometer (Model 8202, JUMO) for remote reading is now available in 52 and 60 mm diameters. Ranges between −60 and +350°C can be supplied. Capillaries in copper or stainless-steel may be up to 15 m long and can be fitted with plain probes, screw fittings, pockets or fast-response coiled bulb. IP51 protection against splash-water is standard, and a 2% accuracy is achieved over the full linear scale. Prices start from £8·15 for 100 units.

*Contact JUMO at The Maltings, Station Road, Sawbridgeworth, Hertfordshire CM21 9JX, UK (tel.: 0279 725501) for more information.*

**Circle No. 162 on Reader Enquiry Card**

### AA pyrolytic cuvette

Pye Unicam has announced a breakthrough in atomic absorption spectroscopy with a new graphite tube for its furnace range, which overcomes the problems of porosity and carbide formation experienced in the past. Called the TPC (Totally Pyrolytic Cuvette), the new tube achieves low porosity and low carbide formation giving a longer analytical lifetime than existing tubes. It also offers the benefits of constant performance and better precision over the lifetime. Previously, graphite tubes of two types have been available—the standard electrographite tubes, suitable for such volatile elements as Pb and Cd and the pyrolytically-coated electrographite tube, used for the less volatile and refractory elements like V, Mo, etc. Coated tubes provide the analyst with the ability to determine the refractory elements at much lower concentration levels, but their surface coating wears down with use, exposing the substrate material so that the analytical performance deteriorates. A solution was to eliminate the substrate altogether and make the tube completely from pyrolytic graphite. Using existing methods it was not possible to make layers thicker than 50 µm, but now Philips has developed an extension of its coating technology enabling it to manufacture Totally Pyrolytic Cuvettes which overcome the residual problems at reasonable cost.

*Further information available from Pye Unicam about the TPC includes independent proof material: Pye Unicam Ltd, York Street, Cambridge CB1 2PX, UK. Tel.: 0223 358866.*

**Circle No. 163 on Reader Enquiry Card**

### Amino-acid analyser

Chromospek M is the latest version of Higer's Chromaspek series. It retains many of the earlier models' features but includes a computer system. It consists of a chromatography system, an Epson QX10 computer with twin floppy disks, a VDU, a graphics printer, an intelligent interface and control and integration software. The computer controls the buffer gradient, sampling signals, column temperature, data capture, storage and calculation functions. Up to 10 buffer gradient profiles can be generated by the operator, stored on disk and used in any order during a single run of samples. Chromatograms are displayed on the screen and printed with peak numbers, retention time, base-line and stop/start integration marks. Each floppy disk has 320K memory; one for system storage, the other for the storage of raw and final data for later analysis. A five megabyte hard disk is available for additional storage.

*More information from Hilger Analytical Ltd, Westwood, Margate, Kent CT9 4JL, UK. Tel.: 0843 25131.*

**Circle No. 164 on Reader Enquiry Card**

### Micro application pump

The latest Model 114M HPLC pump from Beckman features what is considered to be the first 'on-demand' microflow capability for exceptional reproducibility in micro applications. Flow rates are available from 0·001 to 0·999 ml/min, adjustable in increments of 0·001 ml/min. The Model 114M, compatible with all Beckman Series 340 systems, is in the form of a single piston, rapid refill pump. Its simple design improves performance and reliability over multi-piston pumps and the direct torque drive system eliminates gears and permits smooth delivery of solvent.

An intelligent liquid head provides feedback of information for entirely automatic compressibility compensation and 'remembers' proper pressure limits and flow-rate range for both analytical and preparative applications. The 114M has a long-lasting durable seal and digitally-selectable upper and lower pressure limits automatically protect the pump from over-pressurization or loss of pressure. A battery back-up is standard.

*Details from Beckman-RIIC Ltd, Progress Road, Sands Industrial Estate, High Wycombe, Buckinghamshire, UK. Tel.: 0494 41181.*

**Circle No. 165 on Reader Enquiry Card**

### Floppy disk for computing integrator

A floppy disk option is now available for the CI-10 computing integrator, giving up to two megabytes of fast access storage (depending on drive type used) and extended capabilities. The option allows unattended storage (archival) of a series of chromatographic runs. All files resident in the CI-10's internal memory can be saved (methods [analyses], peaks and slices). Slices files can be saved 'on the fly', allowing files much larger than the CI-10's internal memory (32KB) to be stored. The chromatographic data can be later reprocessed directly from the disk.

Operation of the disk software is simple, with all interaction between CI-10 and disk presented on the 16-character alphanumeric display. Dialogue is initiated with a single key. The CI-10 will operate with a range of Commodore Business Machines (CBM) disk drives including:

- Model 2031—single drive, 170KB storage
- Model 1001—single drive, 1 megabyte storage
- Model 8050—dual drive, 1 megabyte storage (0·5 megabyte per drive)
- Model 8250—dual drive, 2 megabyte storage (1 megabyte per drive).

*More information from Laboratory Data Control (UK) Ltd, Milton Roy House, High Street, Stone, Staffordshire ST15 8AR, UK. Tel.: 0785 813542.*

**Circle No. 166 on Reader Enquiry Card**

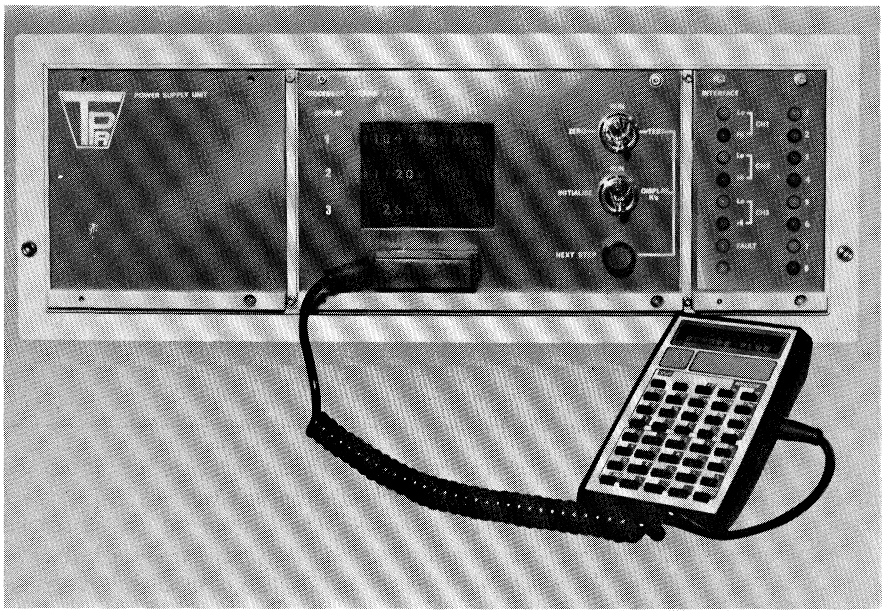

*TPA's microprocessor unit with plug-in keypad. It is suitable for use with most of the company's instruments: the 110 Series Ultra-Violet/Visible Photometers, the 202 Series ATR Analysers, the 230 Series Dust Monitors, the 312 Series Infra-Red Photometers, and the 330 Series Across-Stack Infra-Red Analysers for example.*

### Microprocessor for TPA instruments

A microprocessor unit has been announced by Telsec Process Analysers: it is designed to interface with all of the organization's analysers. The unit gives a continuous digital read-out on up to three channels of concentrations in a sample stream, based on any constants required. It is fully user programmable and, via a plug-in keypad, the sample information can be converted to customer defined parameters, linearization and changes in secondary algorithms.

In addition to standard input and output channels, the unit contains a number of options for customer interfacing. These include analogue channels for sensors to measure temperature, pressure and other variables, and a number of digital input and output channels, which can take logic commands to and from the main process or ancillary instrumentation. The unit has an RS232 interface and three analogue channels, which can be assigned by software to transmit the data obtained in a normal cycle of operation.

The microprocessor has an on-line diagnostic feature. In its normal running mode it determines whether or not the information being obtained from the analyser is of a sufficiently high quality to provide a reliable answer to the measurement being carried out. If it is not, an alarm indicator is illuminated.

Another feature is the ability of TPA to diagnose faults by a telephone link with the microprocessor on the customer's premises. By a modem connection with a base computer at TPA's service centre, an engineer can run a test program to examine the analyser.

*Details from Telsec Process Analysers Ltd, 34 Tresham Road, Orton Southgate, Peterborough PE2 0SE, UK. Tel.: 0733 235500.*

**Circle No. 167 on Reader Enquiry Card**

### High-temperature DSC

DSC 1500, a high-temperature differential scanning calorimeter, from Stanton Redcroft operates from ambient temperature to 1500°C.

The unit features a platinum-rhodium DSC plate with positive crucible location for good calorimetric precision. The plate is mounted in a high-purity alumina cup and excellent atmosphere control is obtained by passing the desired gas directly through this cup. Samples are typically housed in crucibles 6 mm diameter and 4 mm high, which are available in a variety of materials including platinum, alumina and quartz. The head design also enables crucibles 20 mm high and over to be used, thus eliminating the problems associated with samples that bubble or creep at high temperatures. Good atmosphere can still be maintained by the second standard gas purging system, when the gas passes directly through the furnace chamber.

Scanning at rates from 0·1 to 50°C/min is performed by a low mass water-cooled furnace. An automatic handling system is provided for precision location of furnace and at the end of the experiment, if programmed cooling is not required, the furnace is lifted and cooled automatically using the in-built fan. The furnace temperature is monitored so that the lifting process always takes place at a sufficiently low temperature to avoid thermal shock. The system enables the time between runs to be minimized, thus increasing the run throughput.

A microprocessor-based linearizer module, used in conjunction with a high sensitivity DC amplifier, gives a DSC signal with constant calorimetric sensitivity over the operating range of the instrument. In addition, the linearizer provides cold-junction compensation and enables sample temperatures to be displayed and recorded to 0·1°C.

A chromel plate DSC head is also available. This can be simply plugged-in in place of the standard head and offers all the features of the high-temperature head with increased sensitivity over the range ambient to 700°C.

*Further details from Stanton Redcroft Ltd, Copper Mill Lane, London SW17 0BN.*

**Circle No. 168 on Reader Enquiry Card**

